# Prognostic Value of Brachial–Ankle Pulse Wave Velocity in the Prediction of Cardiovascular Events: Comparison with Brachial Pulse Pressure

**DOI:** 10.3390/jcm14248724

**Published:** 2025-12-09

**Authors:** Bo Kyung Jeon, Hack-Lyoung Kim, Kyung-Jin Kim

**Affiliations:** 1 Department of Cardiology, Ewha Woman’s University Mokdong Hospital, Ewha Woman’s University College of Medicine, Seoul 07985, Republic of Korea; 2 Department of Cardiology, Boramae Medical Center, Seoul National University College of Medicine, Seoul 03080, Republic of Korea

**Keywords:** brachial–ankle pulse wave velocity, brachial pulse pressure, major adverse cardiovascular and cerebrovascular events, prognostic factor

## Abstract

**Background**: Although brachial–ankle pulse wave velocity (baPWV) has been used to predict cardiovascular events, studies comparing it with brachial pulse pressure (brPP) for predictive value have been lacking. We investigated how brPP and baPWV differ in their ability to predict future cardiovascular events. **Methods**: We retrospectively reviewed the clinical data of 11,703 consecutive patients where brPP and baPWV measurements had been made. The primary endpoint was differences in the incidence of major adverse cardiovascular and cerebrovascular events (MACCE). **Results**: Participants had a median age of 61 years, and men accounted for 57.7% of the cohort. During a median follow-up duration of 3.64 years, 347 (3.0%) MACCE occurred. Using established reference values of baPWV > 1800 cm/s and brPP > 60 mmHg, we, respectively, stratified patients by these values. Kaplan–Meier survival curve analysis revealed that both high baPWV and brPP groups displayed elevated MACCE incidence, all-cause mortality, and cardiovascular mortality. After controlling for potential confounders, multivariate Cox regression analysis showed that individuals with elevated baPWV had higher rates of MACCE, overall mortality, and cardiovascular death, whereas brPP was not significantly associated with these outcomes. Subgroup analysis showed a consistent difference in MACCE incidence across all subgroups when stratified by baPWV; however, the significance disappeared in several subgroups when stratified by brPP. **Conclusions**: baPWV exhibited a stronger association with MACCE incidence than brPP. Thus, baPWV may be a more effective factor than brPP for cardiovascular risk stratification.

## 1. Introduction

Arterial stiffness is a well-recognized pathophysiological contributor to cardiovascular disease [1]. Progressive loss of vascular elasticity caused by chronic mechanical stress leads to arteriosclerosis, which represents one of the earliest structural and functional changes in the arterial wall [2]. Accordingly, the assessment of arterial stiffness plays an important role in predicting and preventing cardiovascular disorders.

Pulse pressure (PP) is a well-established measure of arterial stiffness and has been associated with adverse cardiovascular outcomes, including mortality, myocardial infarction, and stroke [3,4,5]. Pulse wave velocity (PWV) is another established indicator of arterial stiffness and has more recently emerged as an independent predictor of cardiovascular events and mortality [6]. Among various PWV measurement methods, brachial–ankle PWV (baPWV) is particularly common in East Asia because it is simple, automated, and shows good correlation with aortic PWV [7,8]. Although numerous studies have highlighted baPWV as an effective predictor of cardiovascular events, few have directly compared its prognostic value with that of PP in real-world populations. Therefore, in this study, we compared the predictive value of baPWV and brachial PP (brPP) for major adverse cardiovascular and cerebrovascular events (MACCE).

## 2. Methods

### 2.1. Study Population

This single-center retrospective study included 12,733 consecutive patients who underwent both baPWV and brPP measurements at Boramae Medical Center between October 2008 and June 2018 [9]. Most of the study participants were regular attendees at the respective hospital, and baPWV and brPP were measured as a part of a cardiovascular health examination. We excluded individuals who met any of the following conditions: unstable hemodynamic status; persistent or recurrent chest discomfort or dyspnea; clinically relevant arrhythmias that were not adequately controlled; an ankle–brachial index outside the range of 0.9 to 1.4; valvular heart disease exceeding mild severity; pericardial effusion; or insufficient clinical data for analysis. After applying these criteria, a total of 11,703 participants were included in the study. Approval for the study protocol was obtained from the Institutional Review Board of Borame Medical Center (Seoul, South Korea), which also granted a waiver of informed consent owing to the retrospective design and use of routinely collected clinical information.

### 2.2. Data Collection

Hypertension was identified when participants had a documented history of the condition, were taking antihypertensive medications, or exhibited persistently elevated blood pressure with systolic values ≥140 mmHg or diastolic values ≥90 mmHg. Blood pressure was recorded from the right arm after the participant had remained seated at rest for at least five minutes. Measurements were obtained by trained nursing or medical staff using an oscillometric device, and the average of several readings was used. Systolic and diastolic pressures corresponded to the first and fifth Korotkoff sounds. Brachial pulse pressure was calculated as systolic minus diastolic blood pressure. Diabetes mellitus was determined based on a previous clinical diagnosis, the current use of glucose-lowering therapy, or fasting plasma glucose levels ≥126 mg/dL on repeated testing. Dyslipidemia was recorded when individuals had a known diagnosis, were receiving lipid-modifying medications, or demonstrated low-density lipoprotein (LDL) cholesterol concentrations >160 mg/dL. Participants who reported regular tobacco use within the prior year were classified as smokers. Obesity was defined as a body mass index (BMI) ≥ 25 kg/m^2^ [10]. Coronary artery disease was considered present if there was a history of myocardial infarction or prior coronary revascularization. Stroke was defined as an acute neurologic deficit attributed to cerebral infarction or intracranial hemorrhage confirmed through neuroimaging. Information on concomitant cardiovascular medications—including antiplatelet agents, calcium channel blockers, beta–blockers, renin–angiotensin system inhibitors, and statins—was also extracted. After an overnight fast, venous blood samples were collected, and laboratory measurements included white blood cell count, hemoglobin, glucose, creatinine, total cholesterol, LDL cholesterol, high-density lipoprotein (HDL) cholesterol, and triglyceride levels.

### 2.3. baPWV Measurements

baPWV was measured using previously described methods [11,12]. On the day of testing, participants were instructed to abstain from smoking, alcohol consumption, and caffeinated beverages such as coffee. Regularly prescribed cardiovascular medications, however, were continued. The measurement was performed under stable conditions after more than 5 min of rest. A volume–plethysmographic apparatus (VP–1000; Colin Co., Ltd., Komaki, Japan) was used for baPWV measurements. Electrodes for electrocardiography were attached to both wrists, and a phonocardiographic sensor was positioned along the sternal border to record heart sounds. Pneumatic cuffs were placed on the upper arms and ankles. PWV was derived by dividing the estimated path length by the measured transit time. The distance between the brachial and ankle sites was calculated from the patient’s height. Transit time was defined as the interval between the initial upstroke of the brachial pulse wave and that of the ankle pulse wave. For analysis, we used the average value obtained from measurements on both sides. Inter-observer reproducibility for baPWV assessment demonstrated a coefficient of variation of 5.1% [12].

### 2.4. Clinical Outcomes

The primary endpoint of this study was the occurrence of major adverse cardiovascular and cerebrovascular events (MACCE) during follow-up. MACCE encompassed cardiovascular death, nonfatal myocardial infarction, coronary revascularization, and stroke [13]. Cardiovascular death included mortalities due to acute myocardial infarction, heart failure, malignant arrhythmias, or other cardiovascular causes, and unexplained sudden death was also classified as cardiovascular in origin. Nonfatal myocardial infarction was identified based on compatible symptoms, characteristic electrocardiographic changes, elevated cardiac biomarkers such as troponin, and confirmatory coronary angiographic findings. Coronary revascularization was defined as undergoing percutaneous coronary intervention or coronary artery bypass grafting. Stroke was diagnosed by neurologists on the basis of neuroimaging findings together with acute neurologic deficits.

### 2.5. Statistical Analysis

Categorical variables were summarized as frequencies with corresponding percentages, and group differences were evaluated using either the chi-square test or Fisher’s exact test. For continuous variables, data were described using means with standard deviations, and comparisons between groups were carried out with Student’s *t*-test or the Wilcoxon rank–sum test, depending on distributional assumptions. Survival analyses were performed using Kaplan–Meier methods, and differences in event-free survival were assessed with the log-rank test. Hazard ratios (HRs) and 95% confidence intervals (CIs) were estimated using Cox proportional hazards models. The proportional hazards assumption was assessed by visual inspection of log–minus–log survival plots and tested using Schoenfeld residuals. Variables included in the multivariable models were selected based on statistical significance in univariable analyses (*p* < 0.05). The following covariates were entered into the final model: age, sex, hypertension, diabetes mellitus, dyslipidemia, smoking, coronary artery disease, use of antiplatelet drugs, calcium channel blockers, beta-blockers, renin–angiotensin system blockers, and statins, as well as white blood cell count, hemoglobin level, estimated glomerular filtration rate, total cholesterol, and high-density lipoprotein cholesterol. Multicollinearity among the covariates included in the multivariable Cox models was evaluated using variance inflation factors (VIFs) in a linear regression framework. All VIF values were below commonly accepted thresholds (<10), indicating no significant collinearity. All analyses were two-tailed, and statistical significance was accepted at *p* < 0.05. Statistical analyses were performed using SPSS version 25 (IBM Corp., Armonk, NY, USA) and R software version 4.3.2.

## 3. Results

Baseline demographic and clinical features of the participants are presented in Table 1. The mean age of the study participants was 61 years, and 57.7% of subjects were men. Regarding the risk factors associated with our patient cohort, approximately half of the patients had hypertension, dyslipidemia, and obesity, and approximately one-quarter had diabetes. Among the study participants, 18.2% were current smokers, 25.8% had a history of coronary artery disease, and 0.5% had a history of stroke. The mean values of our laboratory findings were within the normal range.

We used the established reference values of 1800 cm/s and 60 mmHg for baPWV and brPP, respectively, to predict cardiovascular risk [14,15]. When the patients were divided into groups based on these reference values, the high baPWV group (>1800 cm/s) was older (69.9 vs. 58.1 years, *p* < 0.001) and had a lower proportion of men (51.1% vs. 59.7%, *p* < 0.001) compared with the low baPWV group (Table 2). The patients in the high baPWV group also had a lower BMI. In terms of cardiovascular comorbidities, individuals in the high baPWV group showed higher proportions of hypertension, diabetes mellitus, dyslipidemia, coronary artery disease, and stroke. There was a lower proportion of smokers in the high-baPWV group. Moreover, patients in the high baPWV group tended to take significantly more cardiovascular medications, including antiplatelet drugs, statins, and anti-hypertensive drugs, but not calcium channel blockers. The high baPWV group had significantly higher WBC counts and lower hemoglobin, estimated glomerular filtration rate, total cholesterol, LDL, and HDL values than the low baPWV group; however, the mean laboratory test values of both groups were within the normal range (WBC 4.0–10.0 × 10^3^/µL, hemoglobin 12–18 g/dL, estimated GFR > 60 mL/min/1.73 m^2^, total cholesterol 125–200 mg/dL, LDL cholesterol 0–160 mg/dL, and HDL cholesterol 40–60 mg/dL). When patients were divided into groups based on their brPP values, the overall data trends were similar to those of the baPWV groups. However, regarding BMI, patients in the high brPP group (>60 mmHg) had significantly higher BMI values (25.2 vs. 24.7 kg/m^2^, *p* < 0.001), whereas those in the high baPWV group had significantly lower BMI values (24.4 vs. 25.0 kg/m^2^, *p* < 0.001). In addition, the significant differences observed in the baPWV-based groups relating to history of stroke, beta–blocker administration, WBC counts, and HDL levels were not observed in the brPP-based groups. Conversely, members of the high brPP group used significantly more calcium channel blockers than those in the low brPP group, and these differences were not seen in the baPWV-based groups.

**Figure 1:**
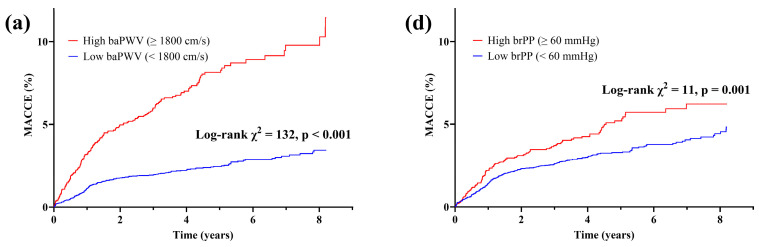

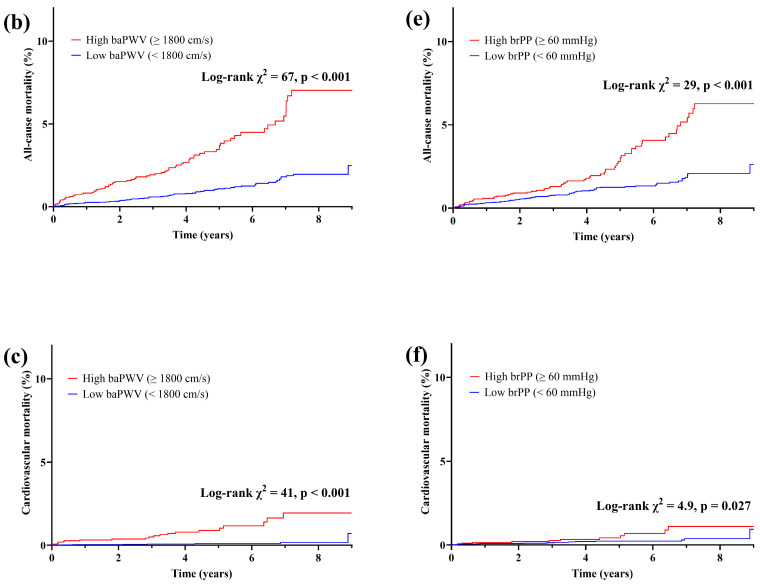
Kaplan–Meier curves showing the cumulative incidence of MACCE, all-cause mortality, and cardiovascular mortality according to high versus low baPWV (≥1800 vs. <1800 cm/s; panels (**a**–**c**)) and high versus low brPP (≥60 vs. <60 mmHg; panels (**d**–**f**)). Log-rank χ^2^ and *p*-values are presented in each panel. baPWV, brachial–ankle pulse wave velocity; brPP, brachial pulse pressure; MACCE, major adverse cardiac and cerebrovascular events.

During a 3.64-year median follow-up period (interquartile range, 1.56–5.39 years), 347 (3.0%) cases of MACCE were recorded. Results of the Kaplan–Meier survival curve analysis revealed that both high baPWV and brPP patient groups were significantly associated with an increased MACCE incidence (log-rank *p* < 0.001 for each) (Figure 1a,d) compared to low baPWV and brPP groups. However, the χ^2^ value was higher in the baPWV-based groups compared with those based on the brPP reference value (132 vs. 11, respectively). Regarding all-cause and cardiovascular mortality, both the high baPWV and high brPP groups showed an increased cumulative incidence; however, the χ^2^ values remained higher in the baPWV-stratified groups (67 vs. 29 and 41 vs. 4.9, respectively) (Figure 1b vs. Figure 1e; Figure 1c vs. Figure 1f). In multivariate Cox regression analysis, patients in the high baPWV group were significantly higher risks of MACCE (adjusted HR 2.76; 95% confidence interval (CI) 2.14–3.57; *p* < 0.001), all-cause mortality (adjusted HR 1.89, 95% CI 1.23–2.90; *p* = 0.004), and cardiovascular mortality (adjusted HR 4.90, 95% CI 1.91–12.50; *p* = 0.001) (Table 3). Patients in the high brPP group exhibited a significantly higher incidence of MACCE, all-cause and cardiovascular mortality before adjusting for potential confounders, whereas no such difference was observed after adjusting for potential confounders.

**Figure 2:**
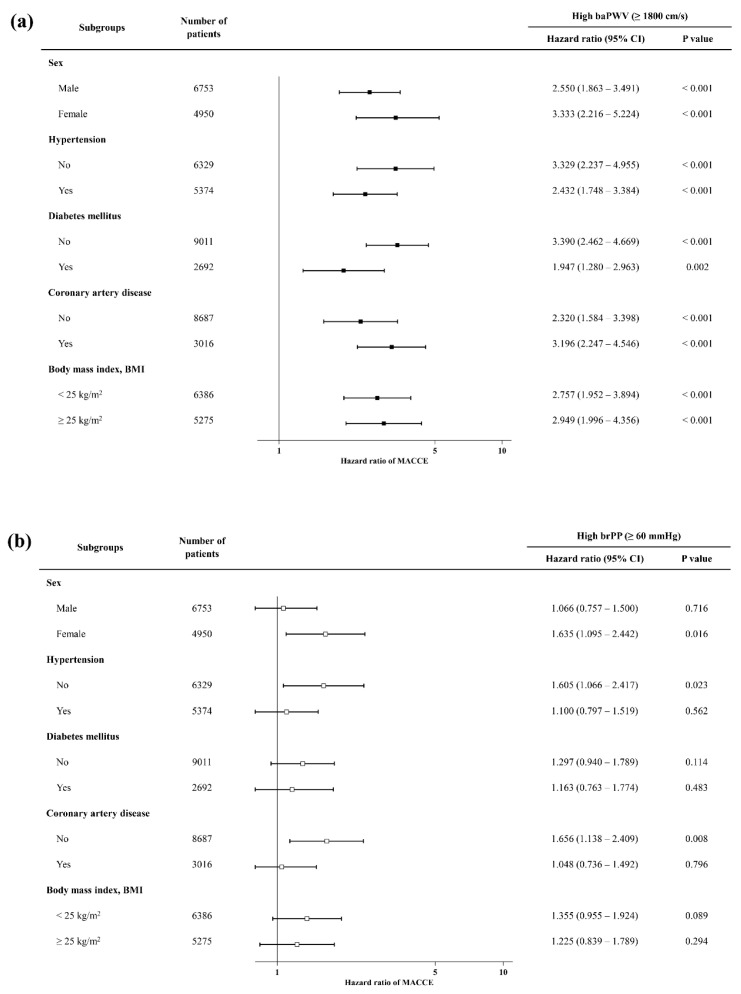
Subgroup analysis. Adjusted hazard ratios for major adverse cardiac and cerebrovascular events (MACCE) were compared between high and low baPWV groups (panel **a**) and between high and low brPP groups (panel **b**) across multiple subgroups. baPWV, brachial–ankle pulse wave velocity; brPP, brachial pulse pressure; CI, confidence interval.

After stratifying the participants into subgroups based on sex and representative cardiovascular risk factors, the hazard ratio values for baPWV and brPP were examined within each subgroup. When the participants were grouped by using the baPWV reference value, a consistently significant difference in the incidence of MACCE (higher hazard ratio) was observed across all subgroups (Figure 2a). However, when the participants were divided using the reference value of brPP, these statistically significant differences disappeared in several subgroups, such as male sex, hypertension, and previous coronary artery disease (Figure 2b).

## 4. Discussion

In this study, using a large-scale registry, we demonstrated that baPWV had a more robust association with MACCE and its components than brPP. Both the high baPWV and high brPP groups showed similar baseline differences and higher incidences of MACCE and mortality compared with their respective low groups; however, after adjustment for confounders, the prognostic impact remained significant only in the baPWV-based groups. In addition, baPWV showed consistently significant hazard ratios for MACCE across all subgroups, whereas some brPP subgroups did not demonstrate consistent associations. Although derived from a single center and an ethnically homogeneous population, this cohort still reflects a wide range of clinical conditions frequently encountered in practice. This broad inclusion enhances the generalizability of our findings and provides a more comprehensive comparison of these two markers across multiple clinically relevant outcomes.

The reference values of baPWV and brPP for MACCE prediction used in our cohort were 1800 cm/s and 60 mmHg, respectively. Regarding baPWV, Yiming et al. suggested that in an Asian population, the normal baPWV ranged from 1390 to 2120 cm/s [16]. In addition, in many studies targeting patients with various risk factors such as hypertension or coronary artery disease, the baPWV reference value used for predicting cardiovascular and cerebrovascular events generally falls within the range of 1550–1800 cm/s [2,17,18,19]. In this study, we used the reference value of 1800 cm/s as proposed by the Korean Society of Hypertension in 2018, and this falls within the range of the previously mentioned values, reflecting the universality of this value [14]. For brPP, a reference value of 60 mmHg is an established threshold that is known to be a risk factor for cardiovascular disease when exceeded [4,20,21]. We focused on risk stratification using guideline-based thresholds, which aligns with the clinical context in which both baPWV and brPP are typically interpreted.

When participants were divided into high and low groups based on the reference values for baPWV and brPP, both high groups showed a higher prevalence of cardiovascular risk factors than the corresponding low groups. Traditional atherosclerotic risk factors, including diabetes mellitus, dyslipidemia, smoking, and hypertension, are well known to influence baPWV. These shared factors likely contributed to the higher crude incidences of MACCE, all-cause mortality, and cardiovascular mortality observed in both high baPWV and high brPP groups before adjustment for confounders [22,23]. Numerous studies have shown that high baPWV is associated with increased risks of MACCE, all-cause mortality, and cardiovascular mortality across a wide range of populations, from general community cohorts to specialized high-risk groups such as post-myocardial infarction patients [6,17,24,25,26,27,28,29]. PP has also been consistently reported as an independent risk factor for cardiovascular events, including mortality [4,5,30,31]. However, despite extensive evidence for each parameter individually, few studies have directly compared the prognostic value of baPWV and PP.

Consistent with our results, several previous studies have also demonstrated the stronger predictive performance of baPWV compared with PP, although each was conducted in more narrowly defined populations. Lu et al. (2018) showed the superiority of baPWV in a large community-based Chinese cohort, while Cao et al. (2023) reported that increased PP did not affect all-cause mortality among individuals with normal baPWV in another community population [32,33]. Additionally, Eguchi et al. (2020) found that the association between brPP and cardiovascular events was present only within a limited PP range in treated hypertensive patients [34]. While prior studies focused on specific populations, our findings extend these observations by confirming the stronger association of baPWV with MACCE in a broader clinical setting. One plausible explanation is that baPWV reflects both central and peripheral arterial stiffness, whereas brPP primarily captures central arterial stiffness [33,35,36,37]. Because peripheral arterial stiffness has recently been recognized as an important contributor to cardiovascular risk, this broader physiologic representation may partially explain the stronger association between baPWV and MACCE [38]. In addition, brPP in our study was measured non-invasively using a brachial cuff, which is known to underestimate pulse pressure compared with intra-arterial measurements, particularly at higher PP values [39,40]. Such underestimation may have attenuated the observed association between brPP and MACCE relative to baPWV, and this methodological difference should be considered when interpreting the comparative results.

In the subgroup analysis, baPWV consistently exhibited a significant positive hazard ratio for MACCE across various subgroups, whereas brPP showed a lower hazard ratio than baPWV in all subgroups and a loss of significance in several subgroups. Previous studies that reported a weaker association between PP and the extent of coronary artery disease in male versus female patients may be a possible explanation for the loss of significance of PP for MACCE in our study among male patients [41]. In the case of the hypertension and coronary artery disease subgroups, it is plausible that individuals with these conditions in our study, who were already undergoing medical follow-up, had a higher likelihood of consistent use of anti-platelet and anti-hypertensive drugs. This factor may have acted as a confounding variable, potentially contributing to the diminishing prognostic significance of brPP, which has a relatively weaker association with MACCE.

Using a large and heterogeneous real-world database, our study demonstrated that baPWV showed a consistently stronger association with MACCE than brPP across multiple analytical approaches. However, this study has several limitations. First, it was conducted at a single center within a single ethnic population. Because baPWV is predominantly used in East Asian clinical practice and less widely implemented in Western healthcare systems, the applicability of our findings to non-Asian populations may be limited. Future validation in ethnically and geographically diverse cohorts will be necessary to confirm the generalizability of these results. Second, although our cohort included more than 10,000 individuals, it consisted of patients undergoing routine checkups at one medical center and therefore may not fully represent the general population. Participants in our cohort exhibited slightly higher prevalence of cardiovascular risk factors—including hypertension, diabetes, dyslipidemia, and obesity—than those reported in the general Korean population [42]. Nonetheless, the cohort encompassed a broad range of comorbidities without restrictions on specific risk profiles, which may better reflect the real-world population in whom cardiovascular risk assessment is clinically relevant. Finally, our study was retrospective in nature and relied on medical records, which limited detailed analyses of outcomes not explicitly documented (e.g., non-ischemic cardiovascular events) and leaves the possibility of residual confounding despite multivariable adjustment. Future prospective studies will be essential to further validate and strengthen these findings.

## 5. Conclusions

In conclusion, baPWV showed a stronger association with MACCE compared with brPP, both in a large-scale real-world population and across various subgroup populations. Therefore, baPWV may have better prognostic value for predicting cardiovascular risk in patients presenting with various risk factors encountered in real-world clinical scenarios.

## Figures and Tables

**Table 1:** Baseline characteristics of total study subjects.

Characteristics	n (%) or Average ± SD
Age (year)	60.9 ± 12.1
Male (%)	6753 (57.7)
Body mass index (kg/m^2^)	24.8 ± 3.4
Risk factor	
Hypertension (%)	5374 (45.9)
Diabetes mellitus (%)	2692 (23.0)
Dyslipidemia (%)	6062 (51.8)
Smoking (%)	2130 (18.2)
BMI ≥ 25 kg/m^2^ (%)	5275 (45.1)
Coronary artery disease (%)	3016 (25.8)
Stroke (%)	63 (0.5)
Medication	
Antiplatelet (%)	1835 (15.7)
Calcium channel blocker (%)	1883 (16.1)
Beta–blocker (%)	2875 (24.6)
RAS blocker (%)	4035 (34.5)
Statin (%)	5562 (47.5)
Lab	
White blood cell count (/μL)	7.2 ± 3.3
Hemoglobin (g/dL)	13.6 ± 1.9
Glomerular filtration rate (mL/min/1.73 m^2^)	86.0 ± 25.4
Total cholesterol (mg/dL)	166.6 ± 41.7
LDL cholesterol (mg/dL)	98.8 ± 37.1
HDL cholesterol (mg/dL)	48.6 ± 13.1
Triglyceride (mg/dL)	134.3 ± 95.6
Primary outcome	
MACCE (cardiovascular death, AMI, revascularization, and stroke) (%)	347 (3.0)
All-cause mortality (%)	153 (1.3)
Cardiovascular mortality (%)	30 (0.3)
Acute myocardial infarction (%)	43 (0.4)
Coronary revascularization (%)	223 (1.9)
Stroke (%)	95 (0.8)
TOTAL	11,703 (100)

HDL, high-density lipoprotein; LDL, low-density lipoprotein; MACCE, major adverse cardiac and cerebrovascular events; RAS, renin–angiotensin system; SD, standard deviation.

**Table 2:** Baseline characteristics of study subjects classified by brachial–ankle pulse wave velocity (baPWV) or brachial pulse pressure (brPP). Cut-off values of baPWV and brPP for major adverse cardiac and cerebrovascular events (MACCE) prediction were 1800 cm/s and 60 mmHg, respectively. Values were presented as n (%) or average ± SD.

Characteristics	Low baPWV (n = 8989)	High baPWV (n = 2714)	*p* Value	Low brPP (n = 8991)	High brPP (n = 2712)	*p* Value
Age (yr)	58.1 ± 11.6	69.9 ± 9.0	<0.001	59.2 ± 11.9	66.4 ± 11.1	<0.001
Male (%)	5366 (59.7)	1387 (51.1)	<0.001	5552 (61.8)	1201 (44.3)	<0.001
BMI (kg/m^2^)	25.0 ± 3.4	24.4 ± 3.3	<0.001	24.7 ± 3.3	25.2 ± 3.6	<0.001
Risk factor						
Hypertension (%)	3719 (41.4)	1655 (61.0)	<0.001	3763 (41.9)	1611 (59.4)	<0.001
Diabetes mellitus (%)	1724 (19.2)	968 (35.7)	<0.001	1876 (20.9)	816 (30.1)	<0.001
Dyslipidemia (%)	4530 (50.4)	1532 (56.5)	<0.001	4607 (51.2)	1455 (53.7)	0.029
Smoking (%)	1703 (19.0)	427 (15.7)	<0.001	1764 (19.6)	366 (13.5)	<0.001
Obesity (BMI ≥ 25 kg/m^2^) (%)	4176 (46.6)	1099 (40.8)	<0.001	3974 (44.3)	1301 (48.2)	<0.001
Coronary artery disease (%)	2194 (24.4)	822 (30.3)	<0.001	2273 (25.3)	743 (27.4)	0.029
Stroke (%)	41 (0.5)	22 (0.8)	0.039	45 (0.5)	18 (0.7)	0.385
Medication						
Antiplatelet (%)	1357 (15.1)	478 (17.6)	0.002	1374 (15.3)	461 (17.0)	0.034
Calcium channel blocker (%)	1416 (15.8)	467 (17.2)	0.075	1321 (14.7)	562 (20.7)	<0.001
Beta–blocker (%)	2109 (23.5)	766 (28.2)	<0.001	2179 (24.2)	696 (25.7)	0.136
RAS blocker (%)	2933 (32.6)	1102 (40.6)	<0.001	2947 (32.8)	1088 (40.1)	<0.001
Statin (%)	4153 (46.2)	1409 (51.9)	<0.001	4224 (47.0)	1338 (49.3)	0.033
Lab						
White blood cell count (/μL)	7.1 ± 3.2	7.6 ± 3.66	<0.001	7.2 ± 3.0	7.3 ± 4.0	0.786
Hemoglobin (g/dL)	13.8 ± 1.8	12.9 ± 1.93	<0.001	13.7 ± 1.8	13.0 ± 1.9	<0.001
Glomerular filtration rate (mL/min/1.73 m^2^)	88.5 ± 23.9	77.6 ± 28.1	<0.001	88.1 ± 24.1	78.8 ± 28.1	<0.001
Total cholesterol (mg/dL)	168.3 ± 41.4	161.1 ± 42.4	<0.001	167.9 ± 41.7	162.3 ± 41.6	<0.001
LDL cholesterol (mg/dL)	99.9 ± 36.9	95.2 ± 37.6	<0.001	99.7 ± 37.1	95.7 ± 36.8	<0.001
HDL cholesterol (mg/dL)	48.8 ± 12.9	47.9 ± 13.6	0.003	48.7 ± 13.1	48.3 ± 13.0	0.279
Triglyceride (mg/dL)	134.3 ± 96.1	134.1 ± 93.7	0.921	134.7 ± 99.4	132.8 ± 81.5	0.344

BMI, body mass index; HDL, high-density lipoprotein; LDL, low-density lipoprotein; RAS, renin–angiotensin system.

**Table 3:** The Cox regression analysis table between the high vs. low brachial–ankle pulse wave velocity (baPWV) group and the high vs. low brachial pulse pressure (brPP) group. Unadjusted vs. adjusted (age, sex, hypertension, diabetes mellitus, dyslipidemia, smoking, coronary artery disease, taking antiplatelet, calcium channel blocker, beta–blocker, renin–angiotensin system blocker and statin, and the level of whole blood cell counts, hemoglobin, estimated glomerular filtration rate, cholesterol and, high-density lipoprotein) hazard ratio (HR) was shown.

Factor	Unadjusted HR (95% CI)	*p* Value	Adjusted HR (95% CI)	*p* Value
MACCE				
baPWV (>1800 cm/s)	3.214 (2.603–3.968)	<0.001	2.760 (2.137–3.566)	<0.001
brPP (>60 mmHg)	1.468 (1.168–1.845)	0.001	1.267 (0.982–1.635)	0.069
All–cause mortality				
baPWV (>1800 cm/s)	3.468 (2.256–4.763)	<0.001	1.890 (1.229–2.904)	0.004
brPP (>60 mmHg)	2.344 (1.699–3.235)	<0.001	0.966 (0.627–1.490)	0.966
Cardiovascular mortality				
baPWV (>1800 cm/s)	8.366 (3.829–18.276)	<0.001	4.895 (1.914–12.520)	0.001
brPP (>60 mmHg)	2.236 (1.077–4.642)	0.031	1.297 (0.555–3.035)	0.548

CI, confidence interval; MACCE, major adverse cardiac and cerebrovascular events.

## Data Availability

The patient data used in this study are not publicly available due to privacy concerns. These data are stored in a controlled access facility at Borame Medical Center in Seoul, South Korea, and can be obtained from Dr. Hack-Lyoung Kim upon reasonable request.
